# Ductal Macrophages Predominate in the Immune Landscape of the Lactating Mammary Gland

**DOI:** 10.3389/fimmu.2021.754661

**Published:** 2021-10-20

**Authors:** Chervin Hassel, Blandine Gausserès, Laurence Guzylack-Piriou, Gilles Foucras

**Affiliations:** IHAP, Université de Toulouse, ENVT, Institut National de la Recherche Agronomique et Environnement (INRAE), Toulouse, France

**Keywords:** mammary gland, lactation, immunity, scRNA seq, macrophages

## Abstract

The mammary gland is unique in female mammals. Mammary tissue undergoes development and remodeling during lactation, a stage associated with high susceptibility to bacterial infections, inducing an inflammatory condition called mastitis. Although the immune response of the mammary gland has been the subject of intense research to improve prevention and treatment efficacy, the precise definition of its immune composition at this particular physiological stage is still missing. We combined single-cell RNA-Seq, flow cytometry, and three-dimensional confocal microscopy techniques to characterize the immune landscape of lactating murine mammary tissue. Macrophages dominated the immune cell repertoire and could be subdivided into at least two subsets: ductal and stromal macrophages. Ductal macrophages represented approximately 80% of the total CD45^pos^ immune cells and co-expressed F4/80 and CD11c, with high levels of MHC class II molecules. They were strategically poised below the alveolar basal cells in contact with the myoepithelial cell network. Adaptive T and B lymphocytes were remarkably less numerous at this stage, which could explain the limited efficacy of vaccination against mastitis. These results support the view that new strategies to increase mammary immunity and prevent mastitis should be devised.

## Introduction

Female mammals are characterized by the presence of one or several pairs of mammary glands. These highly specialized glands provide high-quality nutrients and passive immunity to their offspring after delivery. Mammary development is mainly post-natal and post-pubertal under coordinated hormonal control, but also occurs throughout life with extensive tissue remodeling, especially during gestation (late development) and at the end of lactation (involution). Recent studies have underscored the importance of immune cells in the development of mammary tissue, highlighting the central role of macrophages in branching and duct elongation (1–3). Lactation is also the stage of highest predisposition to infection by various types of bacteria, leading to an inflammatory condition called mastitis. These infections are highly frequent and detrimental for gland function. This is particularly true in domestic species raised for milk production, with few available treatments, except currently unwanted antibiotic administration, and current vaccine with poor efficacy. Mastitis is a true economic burden for the dairy industry and also an important cause of pain and suffering for the animals. Better knowledge of the composition and functioning of the mammary immune system during lactation is thus necessary to define new strategies based on improving immunity rather than antimicrobial therapy. Little information is available on the immune composition of mammary tissue, notably during lactation. We thus used a combination of single-cell RNA sequencing, flow cytometry, and three-dimensional confocal microscopy after careful separation of the mammary glands from the nearest lymph nodes to describe the immune landscape of murine mammary tissue at the lactation stage. Here, we show that macrophages are the major cell population of the immune compartment in the lactating mammary gland. Several subsets can be distinguished, and ductal macrophages, located at the lumen interface, represent a majority of the immune cells, whereas adaptive lymphocytes are much less numerous in this tissue.

## Materials and Methods

### Mice

Ten- to sixteen-week-old female C57BL/6 mice (Charles River) were bred and housed in a specific pathogen-free facility (INSERM US 006 – CREFRE). Experiments were performed in an accredited research animal facility of the UMR IHAP, ENVT, Toulouse, France.

Mice were handled and cared for according to the ethical guidelines of our institution and following the Guide for the Care and Use of Laboratory Animals (National Research Council, 1996) and the European Directive EEC/86/609, under the supervision of authorized investigators. Mice were euthanized by cervical dislocation and all efforts were made to minimize animal pain and distress.

### Blood Cell Staining For Exclusion

Mice were intravenously injected with 100 µg CD45 antibody FITC-labeled (clone REA737, Miltenyi Biotec)/PBS solution (Miltenyi Biotec) using a 1 mL insulin syringe. Mice were sacrificed 5 min after injection following a previously described protocol (4).

### Single-Cell Preparation, TotalSeq Staining, and Immune-Cell Isolation

The fourth pair of lactating mammary glands were harvested from the mice and the inguinal lymph nodes carefully removed from the tissue preparation. Each mammary gland was finely sliced and digested with Liberase at 83 µg/mL and DNAse I at 40 µg/mL in HBSS, 0.5% BSA, 10 mM Hepes for 20 min at 37°C. Digested preparations were passed through filters of diminishing grid size (100, 70, and 40 µm), crushed between two filters, and washed three times. The number of cells of each lactating mammary gland preparation were determined, adjusted to 10 x 10^6^ cells per mL, and incubated with FcR Blocking Reagent (Miltenyi Biotec) for 10 min on ice to avoid non-specific labelling through Fc-receptors. Lactating mammary gland cell suspensions of each mouse were labeled using TotalSeq™ anti-mouse hashtag 1-3 antibodies A301-303 (Biolegend, UK) for 30 min at 4°C. After two washing steps, the three suspensions were pooled and stained with anti-IgG2b-Vioblue antibody to specifically label CD45 cells. Cell suspensions were then labeled with 7AAD (Biolegend) as a viability marker. Doublet, 7AAD^pos^ and FITC^pos^ cells were gated out to sort only viable resident CD45^pos^ immune cells using a BD FACS Influx instrument (BD Biosciences) and 90,000 cells were recovered.

### Single-Cell RNA Sequencing Using the 10X Genomics Approach

Cell viability was confirmed using an aliquot of Trypan blue-treated sorted cells. The cells were encapsulated in a water-in-oil emulsion in contact with unique barcode-coated gel beads using the 10X Genomics Chromium technology. Almost 18,000 cells were injected into two lanes of a Chromium controller. Then, the scRNA-Seq library was prepared using Chromium Single Cell 3ʹ Reagent Kits v3 according to the manufacturer’s instructions. The libraries were pooled and sequenced using one lane of an Illumina HiSeq3000 device following the instructions of 10X Genomics, allowing obtention of almost 300 million reads. The single-cell RNA-seq sequencing data are available under the following accession number GSE183919.

### Analysis of sc-RNA-Seq Data

Sorted sequencing reads were converted from BCL2 to FASTQ files and demultiplexed using bcl2fastq converter (v2.20.0.422). Then, the sequences were aligned to the Mm10 mouse genome using STAR, filtered, and normalized and UMI counting and production of the gene/barcode matrix were performed for all libraries (RNA and HTO, TotalSeq hashtag) using CellRanger pipeline software (v 3.0.2). HTO tags were then counted in all HTO libraries using the Python script CITE-seq-Count (v 1.4.2) with TotalSeq Hashtag (Biolegend) parameters, which allows creation of a hashtag count matrix.

Downstream analyses were then performed using R package Seurat v 4.0.0 (5) and R package UMAP (0.2.7.0). RNA and HTO counts were first normalized by log normalization and demultiplexed using the cell hashing function of Seurat. The number of UMIs for singlets, doublets, and negative cells were next determined and used to keep only singlet cells. The top 1,000 most variable features were determined to scale the RNA data and reduce the dimension of the data using Principal Component Analysis (PCA). Then, the first 10 principal components were selected to cluster the cells using the Louvain algorithm and various resolution parameter values. The non-linear dimension reduction UMAP technique was used to visualize and explore the cell clusters. Differentially expressed genes (biomarkers) for each cluster were determined using the non-parametric Wilcoxon rank sum test and heatmap representations. Cell-type attribution for each cluster was performed using both marker gene analysis and automatic cell-type attribution based on R package clustifyr (6). Indeed, clustifyr uses a wide range of prior knowledge of cell types (clustifyrdata R package) to assign general cell identities to each cell cluster in the dataset. Trajectory analysis was performed using the dynverse workflow based on the dyno (v 0.1.2) and tidyverse (v 1.3.0) R packages. This pipeline provides the advantage of comparing more than 50 trajectory inference methods and inferring the trajectory without prior assumptions (7). The slingshot method was selected as the best and used to infer the pseudo-time and trajectory. The trajectory was visualized on the UMAP coordinates using R package dynplot (v 1.0.2.9000). Gene-set enrichment analysis (GSEA) was performed using R package ClusterProfiler v 3.18 (8). This analysis makes it possible to associate the marker genes of each cluster with enriched functions or cellular pathways. ReactomePathwayAnalysis (v 1.34) was used for the GSEA of each macrophage cluster and the compareClusters function to compare differential enrichment across clusters.

### Flow Cytometry

A single-cell preparation was obtained from mammary gland tissue as described above. Cell numbers were determined by the flow cytometry absolute counting system (MACSQuant Analyzer, Miltenyi Biotec, Germany). Cells (1-2 x 10^6^) were incubated in HBSS, 0.5% BSA, 10 mM Hepes containing mouse FcR Blocking Reagent (Miltenyi Biotec, Germany) following the manufacturer’s instructions. Cell viability was assessed using Viobility 488/520 Fixable Dye (Miltenyi Biotec, Germany). Incubation with the antibodies was performed at 4°C for 30 min in the dark. The antibodies used were: Ly-6G VioBlue (REAfinity™, clone REA526, Miltenyi Biotec), CD45 VioGreen (REAfinity™, clone REA737, Miltenyi Biotec), CD335 (NKp46) PE (REAfinity™, clone REA815, Miltenyi Biotec), CD19 PE-Vio 615 (REAfinity™, clone REA749, Miltenyi Biotec), CD11c PE-Vio 770 (REAfinity™, clone REA754, Miltenyi Biotec), F4/80 APC (REAfinity™, clone REA126, Miltenyi Biotec), CD3 APC-Vio770 (REAfinity™, clone REA641, Miltenyi Biotec), Ly-6C VioBlue (REAfinity™, clone REA796, Miltenyi Biotec), Ly-6G FITC (REAfinity™, clone REA526, Miltenyi Biotec), CD335 (NKp46) FITC (REAfinity™, clone REA815, Miltenyi Biotec), CD19 FITC (REAfinity™, clone REA749, Miltenyi Biotec), CD3 FITC (REAfinity™, clone REA641, Miltenyi Biotec), CD206 (MMR) PE (rat, C068C2, Biolegend), CX3CR1 PerCP/Cyanine5.5 (mouse, SA011F11, Biolegend), CD64 APC-Vio770 (REAfinity™, clone REA286, Miltenyi Biotec), and CD11b APC-Vio770 (REAfinity™, clone REA592, Miltenyi Biotec). Acquisition was performed with a MACSQuant (Miltenyi Biotec, Germany) flow cytometer using MACSQuantify software. Flow cytometry data were analyzed using FlowJo (Tree Star, USA) software.

### Confocal Microscopy on Mammary Tissue After Optical Clearing

The anti-mouse primary antibodies used were: mouse IgG2aκ anti-α-smooth muscle actin clone 1A4 (eBioscience, 14-9760-82, 1/500), rat CD45 clone 30F-11 (BioLegend, 103102, 1/200), rabbit F4/80 clone D4C8V (Cell Signaling, 30325S, 1/400), rabbit CD11c clone D1V9Y (Cell Signaling, 97585S, 1/150), rat CD11b clone M1/70 (BioLegend, 101247, 1/500), and rat MHCII clone M5/114.15.2 (BioLegend, 107601, 1/200). The secondary antibodies used were (all diluted at 1/500 and from Invitrogen unless otherwise specified): Alexa Fluor 488 goat anti-mouse IgG2aκ (A-21131), Alexa Fluor 568 goat anti-rat IgG (A-11077), Alexa Fluor 647 goat anti-rabbit IgG (A-21244), and Alexa Fluor 594 goat anti-rabbit Fab fragment (Jackson ImmunoResearch, 111-587-003, 1/160).

The clearing protocol was adapted from Rios et al. (9). Briefly, tissues were fixed in 4% paraformaldehyde, washed in PBS, and incubated overnight with primary antibodies. After several washing steps, tissues were incubated overnight with secondary antibodies, and then DAPI before clearing in FUnGI (10).

Images of the samples were captured using a Zeiss LSM 710 confocal microscope equipped with x20/0.8 PL APO oil differential interference contrast (DIC) and x40/1.3 PL APO oil differential interference contrast objectives using the spectral unmixing mode at 512 x 512 pixels. Images were processed using Zen (Zeiss) and FIJI/ImageJ (11).

## Results

### Single Cell Transcriptomics Reveals the Unbiased Immune Landscape of Mammary Tissue at Lactation Stage

**Figure 1:**
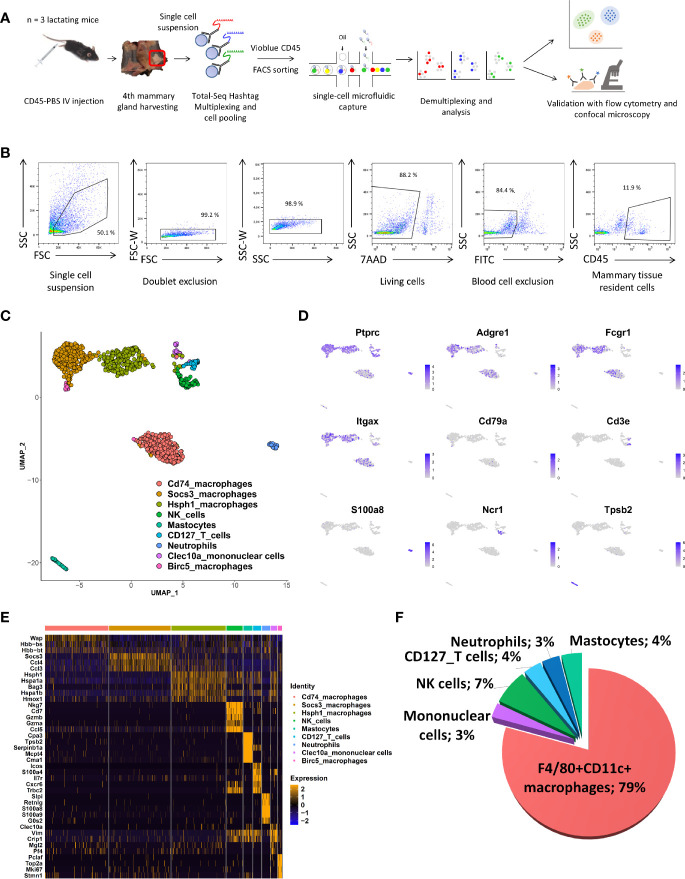
scRNA-Seq description of immune-cell composition of the lactating mammary gland. **(A)** Schematic overview of the experimental pipeline used to perform the10X Chromium scRNA-Seq experiments on mammary tissue from lactating mice (n = 3). **(B)** Gating strategy used for FACS sorting prior to the use of single-cell Gel Beads in Emulsion (GEM). **(C)** UMAP plot of multiplexed CD45^pos^ cells isolated from the lactating mammary glands of two mice. The colors indicate clusters identified after automatic assignation. **(D)** Feature UMAP plots displaying the level of single-cell expression of key cell-type markers. **(E)** Heatmap indicating the five most differentially expressed genes for each CD45^pos^ cell cluster. **(F)** Proportion of CD45^pos^ immune cells in the lactating mammary gland determined by scRNA-seq. The numbers indicate the percentage of each cell type among all immune CD45^pos^ cells.

We set up a single-cell isolation protocol from whole mammary tissue of the fourth pair of glands of lactating C57BL/6 mice using gentle physical trituration and enzymatic digestion to determine the exact resident immune cell composition without any *a priori* assumption. Blood cells were excluded by pre-labelling with FITC-labelled CD45 antibody injected intravenously shortly before euthanasia, as previously described (4). Lymph nodes were also carefully removed during dissection of the mammary glands in order to isolate truly mammary-resident cells, except for one mouse to estimate the impact of any remaining lymphoid tissue on the composition (**Supplementary Figure 1**). After labeling using TotalSeq A hashtag antibodies for multiplexing, CD45^pos^ cells were sorted from a pooled cell suspension after the exclusion of dead and CD45-labeled blood cells. Single-cell RNA-seq was applied to sorted cells of three mice using the protocol of 10X Genomics, as summarized in **Figures 1A, B**. A total of 6,427 unique cells were obtained by Cellranger pipeline analysis and sample demultiplexing using the Seurat pipeline. A proportion of the cells (n = 1,817) showed very low levels of *Ptprc* (CD45), corresponding to endothelial cells. They were excluded from further analysis. At this step, we noted that the sample corresponding to the mammary gland that included the inguinal lymph node had a different cell composition from that of the others (**Supplementary Figure 1**); this mouse was excluded from further analysis. Unbiased clustering and UMAP dimensional reduction algorithms were applied to the remaining cells, resulting in nine clusters (**Figure 1C**). The cell clusters were assigned to a given cell type without any prior assumptions based on combining gene expression levels of known immune markers, as indicated below, and automatic attribution using the *clustifyr* package, (**Figure 1D** and **Supplementary Figure 2**). UMAP patterns showed six different cell types, corresponding to T lymphocytes (*Cd3e*), NK cells (*Ncr1*), neutrophils (*S100a8*), mastocytes (*Tpsb2*), mononuclear cells, and macrophages. Several different clusters of macrophages could be identified based on typical expression patterns (with *Adgre1* and *Fcgr1* as the main genes) and the mRNA levels of *Cd74*, *Socs3*, *Hsph1*, *Clec10a*, and *Birc5* genes (**Figure 1E** and **Supplementary Figure 3**). T cells were recognized as *Cd3e-*, *Il7r-*, and *Cxcr6*-expressing cells, but no *CD4* or *CD8a* expression was detected in this cluster, as observed elsewhere, due to the limited sensitivity of the scRNA-seq technology (12). The chart plot clearly shows that macrophages constitute the main immune cell type in lactating mammary tissue (**Figure 1F**).

### Various Macrophage Subsets Populate the Mammary Tissue

**Figure 2:**
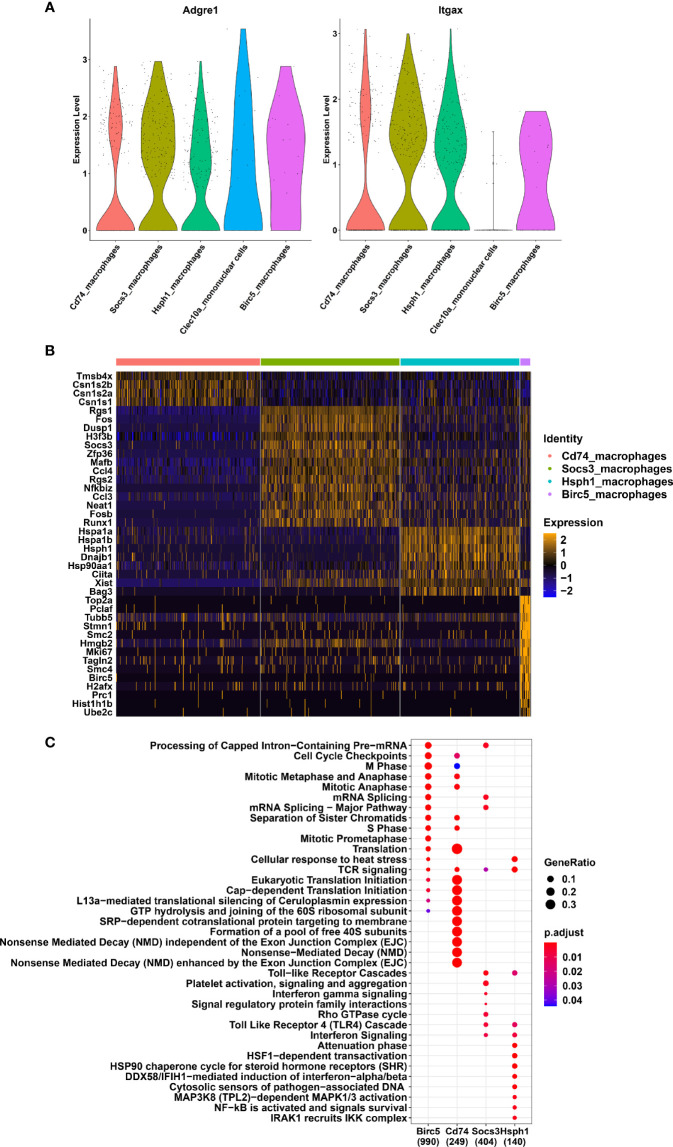
Different subsets of macrophages in the lactating mammary gland **(A)** Violin plots showing the expression of Adgre1 and Itgax genes in mononuclear phagocyte system clusters. **(B)** Heatmap indicating the twenty most differentially expressed genes for each macrophage cluster. **(C)** Reactome pathways enriched in each macrophage cell cluster. The colors of the dots correspond the adjusted p-value. The size of each dot corresponds to the GeneRatio.

We next focused our analysis on the various subsets amongst the macrophage clusters. We excluded the *Clec10a* cluster from the analysis because it corresponds to a mix of dendritic cells (*Zbtb46*) and macrophages. We compare the expression level of Adgre1 (F4/80) and Itgax (CD11c) between all the remaining macrophage clusters (**Figure 2A**). The result clearly showed high expression levels of both markers with a slightly lower *Itgax* levels in *Birc5*_macrophages. Differential expressed genes between the 4 macrophage clusters have been determined and are represented with an heatmap (**Figure 2B**). *Birc5* cluster expressed high levels of genes in cell cycling such as *Top2a*, *Pclaf*, *Stmn1*, *Mki67* and *Birc5* genes. The *Cd74*_macrophages expressed few enrichment genes like *Cd74* (**Supplementary Figure 3**), *Tmsb4x* and some milk-protein transcripts as *Csn1s2b*, *Csn1s2a* and *Csn1s1*. Moreover, this cluster presented lower expression levels of *Ptprc* (CD45), *Adgre1* (F4/80), *Itgax* (Cd11c), and *Fcgr1* (CD64) than the *Birc5* and *Socs3* macrophage subsets (**Supplementary Figure 3**). The *Socs3*_macrophage cluster expressed high levels of classical canonical macrophage markers, such as *Csf1r* (M-CSFR), *Cx3cr1*, and *Cd68* (**Supplementary Figure 3**) and immune functions genes like *Socs3*, *Rgs1*, and *Dusp1* (**Figure 2B**). The *Hsph1*_macrophages were genetically close to the *Socs3*_macrophages (**Figure 1C**) but expressed several different genes, such as the HSP family genes *Hspa1a*, *Hspa1b*, *Hsph1*, *Dnajb1*, *Ciita*, and *Bag3*. We next investigated the cellular functions of each macrophage clusters using a Gene Ontology enrichment analyze (**Figure 2C**). The results clearly indicated a division of cellular functions as *Birc5* cluster is a dividing cell population. *Socs3*_macrophages and *Hsph1*_macrophages are clearly involved in immune functions such as IFNγ signaling and TLR4 Cascade. The second cluster showed others activated signaling pathways as HSP and NF-KB. In addition, we used a trajectory inference approach, without any prior assumption, using the slingshot model in the *Dyno* pipeline (7) to understand the relationship between the macrophage subsets (**Supplementary Figure 4**). The inferred trajectory showed *Birc5*_macrophages as the precursors of the various lineages or subsets, followed by *Socs3*_macrophages. Next, the trajectory splits into *Hsph1*_macrophages and *Cd74*_macrophages.

### Cell Surface Expression by Flow Cytometry Defines Two Main Populations of Mammary Macrophages

**Figure 3:**
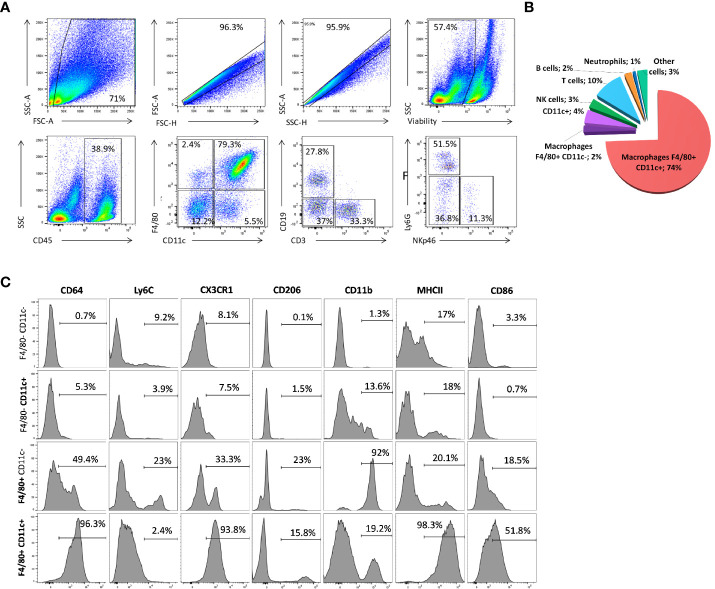
Immune-cell composition of the lactating mammary gland. **(A, C)** Flow cytometry analysis of the immune landscape on single-cell suspension from lactating mammary tissue. The numbers indicate the percentage of cells within the corresponding gate. These patterns are representative of one of 10 individual mice analyzed, each giving similar results. **(B)** Pie chart showing the proportions of CD45^pos^ immune cells in the lactating mammary gland by flow cytometry. The numbers indicate the percentage of each cell type among all living CD45^pos^ immune cells. **(C)** Flow cytometry analysis of the level of various markers in F4/80 CD11c double-negative, single-positive, and double-positive CD45^pos^ cell sub-populations.

To further confirm the scRNA-seq results, we next analyzed the mammary cell composition by flow cytometry using the same protocol to prepare single cell suspensions (**Figure 3A**). Immune cells (identified as CD45^pos^, as before) represented almost 22% of the events recognized as living cells. They were divided amongst macrophages (F4/80^pos^ CD11c^pos^, 79%; CD11c^pos^ single-positive cells, 5.5%), T cells (CD3^pos^, 5%), B cells (CD19^pos^, 3.4%), and NK cells (NKp46^pos^, 0.5%) (**Figure 3B**). Based on the F4/80 and CD11c markers, the macrophages subdivided into two subpopulations, with most (> 90%) included in a continuum expressing both markers (**Figure 3B**). None of the markers identified by scRNA-seq enabled clear separation into the macrophage subsets identified at the transcriptional levels. Indeed, this double-positive population expressed certain other key immune surface markers, such as CD64, CX3CR1, and MHC II molecules (**Figure 3C**). Only 16% expressed CD206, and 52% were positive for CD86. CD11b is expressed in 19% of the double positive cells, but Ly6C was not detected in this macrophage subset (**Figure 3C**). All genetic and molecular markers indicate that this cell population very probably corresponds to recently discovered ductal macrophages (2). On the contrary, high and uniform expression of CD11b was detected in F4/80^pos^ CD11c^neg^ cells and they likely correspond to stromal macrophages, as previously shown (2).

### Double-Positive CD11c^pos^ F4/80^pos^ Mammary Macrophages Are Ductal Macrophages Located Beneath the Mammary Epithelia

**Figure 4:**
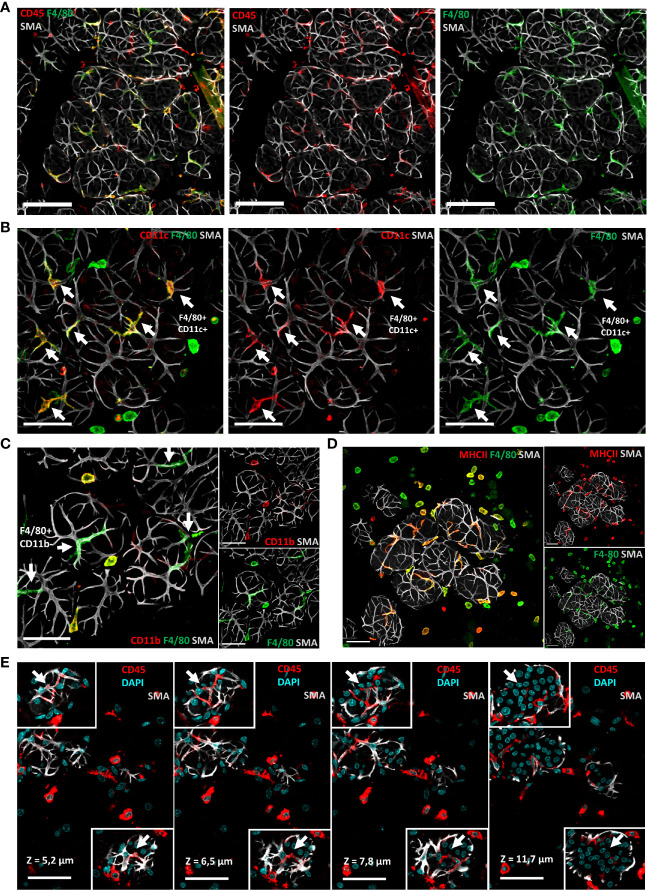
Most macrophages around alveoli have a stellar shape and correspond to so-called ductal macrophages. **(A–D)** Three-dimensional maximum intensity projections (MIPs) of confocal microscopy images representative of optically cleared C57BL/6J lactating mammary glands. **(A)** Predominance of stellar-shaped F4/80 ^pos^ alveolar cells closely associated with SMA^pos^ myoepithelial cells among mammary gland CD45^pos^ leukocytes. **(B–D)** F4/80 ^pos^ alveolar cells (white arrows) are CD11c ^pos^, CD11b^neg^, and MHCII ^pos^. **(E)** Four sequential optical slices (1.3 µm thick), in which the depth (*z* value) is relative to the first image in the sequence, showing the intercalary position of CD45^pos^ CD11c^pos^ cells (white arrows) between the luminal and basal epithelial cells in two distinct alveoli. Scale bars = **(A)** 100 µm, **(B–E)** 50 µm.

To determine the spatial organization of the above-described cell types, notably the macrophage subsets that could be identified on the basis of the markers F4/80 and CD11c, we next examined tissue sections by confocal microscopy, after tissue clearing, using antibodies against both markers. Confocal images showed the presence of CD45^pos^ F4/80^pos^ CD11c^pos^ MHCII^pos^ CD11b^neg^ stellate-shaped alveolar cells, which were closely associated with SMA^pos^ myoepithelial cell network (**Figures 4A–D**). Such localization confirms their definition as ductal macrophages. We also used additional markers for their identification like CD45, F4/80, CD11c, CD11b, CX3CR1, and MHCII (2), (**Figure 4E**) and confirmed previous observations showing that macrophages localize immediately adjacent to alveolar basal cells during lactation, where they frequently imitate basal-cell morphology (13). Macrophages were also present within alveoli, consistent with their enrichment in milk (14).

## Discussion

The exact immune-cell composition during lactation (7 to 10 days after delivery in mice, when lactation is well established) was ill-defined until now (15, 16). In humans, no work using scRNA-seq reported about the immune cell composition of healthy breast tissue, and even more so during lactation. By ontogenesis, the mammary gland is an adipose tissue containing not only adipocytes, but also epithelial, endothelial, and immune cells (17). Immune cells are much less numerous than epithelial, endothelial, and stromal cells. For that reason, enrichment of immune cells using specific markers like CD45 is needed before droplet encapsulation, when a great number of immune cells want to be profiled. Only, Azizi et al. performed sorting on CD45^pos^ immune cells from breast cancer patients undergoing mastectomy before encapsulation (18). As a consequence, most studies describe the epithelial cell composition in tumorigenic micro-environment (19, 20).

### Improvements Over Existing Protocols for Tissue Resident Cell Profiling

Determination of the cell composition is highly dependent on the mode of single-cell preparation and the technical approach used for enumeration. Previous studies considering whole mammary tissue without a pre-enrichment step have examined a low number of immune cells (17, 21). Furthermore, the tissue is highly vascularized at the lactation stage to provide the nutrients necessary for milk synthesis. Immune cells, like neutrophils and monocytes, may also be recruited from the blood into the tissue through diapedesis when an infection occurs. The well-developed vascular network is thus a source of non-resident cells that must be excluded before analysis when the purpose is to describe tissue-resident cells of a healthy gland.

We pay much attention to remove any extra tissue form the single cell suspension to avoid spill over from lymphocyte-rich tissue, whether they are blood or lymph node. The cell composition determined in this work differed significantly from previous reports (17, 21). Only one of our samples, with a draining lymph node included, had a composition similar to that in previously published reports, indicating that previous descriptions of mammary cell composition were biased by the co-isolation of lymphoid tissue at the same time as mammary tissue. The abundance of lymphoid T and B cell is probably due to the presence of one or several lymph nodes that were extracted with mammary tissue, increasing erroneously the proportion of lymphocytes.

High HSP gene expression suggests that *Hsph1*_macrophages may correspond to an activated subset (22). However, we cannot exclude that this gene expression pattern is not biologically relevant and is the consequence of tissue digestion with enzymes at physiological temperature (23). It is technically possible to use alternative protocols for single cell preparation by using low temperature digestion as described recently. This protocol remains to be evaluated in further details for mammary tissue, to see if cell recovery remains optimal and cell activation may be avoided.

### Different Macrophage Subsets Are Present Within the Mammary Tissue and Have a Different Pattern of Distribution

At least two categories of macrophages populate the mammary tissue: ductal and stromal macrophages, that are not only distinguished by different expression of usual surface markers, but also by their location. Within the ductal macrophage subset, different types are also present that may have different functions, states of activation or differentiation stages. Indeed, *Birc5*_macrophages seem to be precursor and dividing cells. This particular cell subset has been previously designated as “stem-like macrophages” in mouse adipose tissue (24). *Socs3*_macrophages could be non-activated macrophages which could quickly respond to different cytokines or detect the presence of pathogens as bacteria in the context of mastitis or play a major role in the mammary gland tissue remodeling. *Hsph1*_macrophages seem to be activated *Socs3*_macrophages because of their genetic closeness in the UMAP representation (**Figure 1C**). Finally, *CD74*_macrophages expressed high levels of MHC II-related genes (*CD74*, *H2-Aa*, and *H2-Eb1*) and milk-protein transcripts (*Csn2*, *Csn1s2a*, *Csn1s2b*). This cluster should correspond to macrophages that phagocytosed epithelial cells, which led to a confusing pattern of gene expression and assignment, as described previously (2). This is in accordance with the high cell turnover, milk exfoliation, and phagocytosis observed in the lactating mammary gland (2). The sensitivity of detection of the scRNA-seq method is often low as the number of differentially expressed genes may not be sufficient to ascertain the functions. So, the exact functions and involvement of each macrophage clusters in the mammary gland remain to be examined in further details.

### Lessons for Mammary Immune Defenses and Further Paths for Improvement

We showed that various sub-populations of macrophages constitute the major immune cell types in lactating mammary glands, conversely to adaptive immune cells that are scarce in the mammary tissue (15). Notably, Betts et al., showed a decreased abundance of total CD4^pos^ cells compared to the nulliparous stage and during involution. These findings are key to understanding how to overcome mastitis and to developing new preventive strategies by improving mammary gland immunity against bacterial infections though vaccination or immune training. Future studies are needed to understand the role of the various subsets of ductal macrophages in the immune homeostasis of the mammary gland and the response to infection.

## Data Availability Statement

The datasets presented in this study can be found in online repositories. The names of the repository/repositories and accession number(s) can be found below: Gene Expression Omnibus database under the following accession number GSE183919.

## Ethics Statement

The animal study was reviewed and approved by Unité IHAP INRAE UMR 1225, Ecole Nationale Vétérinaire de Toulouse - D3155527.

## Author Contributions

CH, BG, LG, and GF designed the experiments, analyzed the data, and wrote the manuscript. CH and BG performed the research and prepared the figures. All authors contributed to the article and approved the submitted version.

## Funding

This work was supported by grants from the Agence Nationale de la Recherche (ANR REIDSOCS; ANR-16-CE20-0010) to GF.

## Conflict of Interest

The authors declare that the research was conducted in the absence of any commercial or financial relationships that could be construed as a potential conflict of interest.

## Publisher’s Note

All claims expressed in this article are solely those of the authors and do not necessarily represent those of their affiliated organizations, or those of the publisher, the editors and the reviewers. Any product that may be evaluated in this article, or claim that may be made by its manufacturer, is not guaranteed or endorsed by the publisher.

## Supplementary Material

Supplementary Figure 1 Comparison of the immune-cell composition of the lactating mammary gland with and without the inguinal lymph node. **(A)** UMAP plot of multiplex CD45 ^pos^ cells isolated from the lactating mammary gland from each of three mice (one including the inguinal lymph node). Cells indicate the assigned clusters. **(B)** Dot plot showing the average expression of the indicated genes and the percentage of cells within each cell cluster of the lactating mammary gland of mouse 2, which included the inguinal lymph node. The colors of the dots correspond to the average expression of the corresponding gene within each cell cluster. The size of each dot corresponds to the percentage of the cells expressing the gene within each cluster. **(C)** Proportion of CD45 ^pos^ immune cells in the lactating mammary gland of mouse 2, which included the inguinal lymph node, determined by scRNA-seq. The numbers indicate the percentage of each cell type among all immune CD45 ^pos^ cells.

Supplementary Figure 2 Automatic single-cell cluster annotation of lactating mammary gland immune cells using the *clustifyr* package and the following reference datasets: Mouse Cell Atlas **(A)**, Tabula Muris **(B)**.

Supplementary Figure 3 Dot plot showing the average expression of indicated genes and the percentage of cells within each cell cluster. The colors of the dots correspond to the average expression of the corresponding gene within each cell cluster. The size of each dot corresponds to the percentage of cells expressing the gene within each cluster.

Supplementary Figure 4 Trajectory inference of CD45^pos^ immune cells in the lactating mammary gland. The trajectory inference was performed using the Dyno package (slingshot model). In the left panel, the colors indicate the identified cluster after automatic assignation and in the right panel the inferred pseudo-time.
