# Supplementary figures and images for: Ductal Macrophages Predominate in the Immune Landscape of the Lactating Mammary Gland
Source: Front Immunol. 2021 Oct 20;12:754661. doi: 10.3389/fimmu.2021.754661 (PMC8564477; doi:10.3389/fimmu.2021.754661)

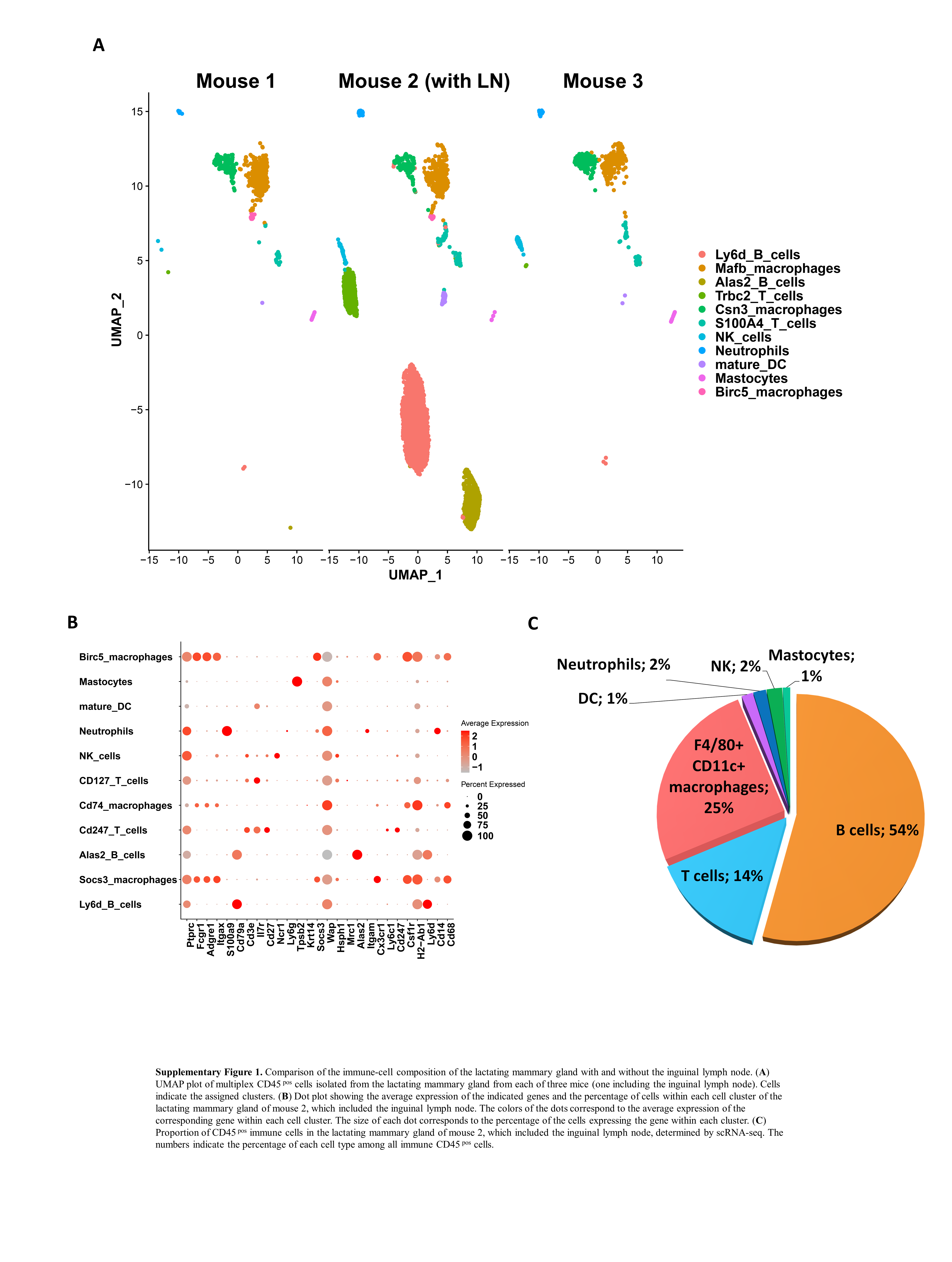

Supplement: Supplementary Figure 1 — Comparison of the immune-cell composition of the lactating mammary gland with and without the inguinal lymph node. (A) UMAP plot of multiplex CD45 pos cells isolated from the lactating mammary gland from each of three mice (one including the inguinal lymph node). Cells indicate the assigned clusters. (B) Dot plot showing the average expression of the indicated genes and the percentage of cells within each cell cluster of the lactating mammary gland of mouse 2, which included the inguinal lymph node. The colors of the dots correspond to the average expression of the corresponding gene within each cell cluster. The size of each dot corresponds to the percentage of the cells expressing the gene within each cluster. (C) Proportion of CD45 pos immune cells in the lactating mammary gland of mouse 2, which included the inguinal lymph node, determined by scRNA-seq. The numbers indicate the percentage of each cell type among all immune CD45 pos cells. [file Image_1.tif]

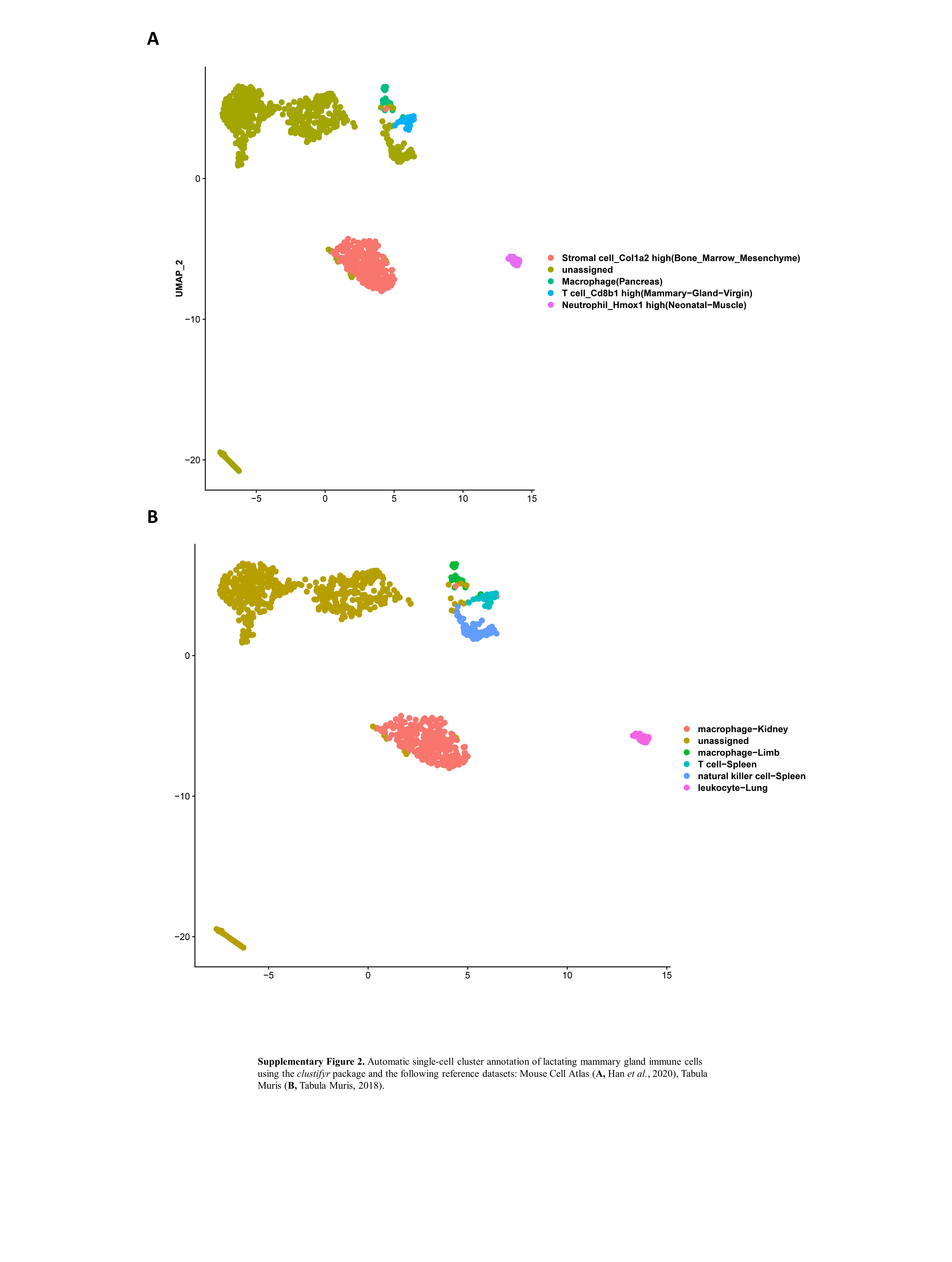

Supplement: Supplementary Figure 2 — Automatic single-cell cluster annotation of lactating mammary gland immune cells using the clustifyr package and the following reference datasets: Mouse Cell Atlas (A), Tabula Muris (B). [file Image_2.tif]

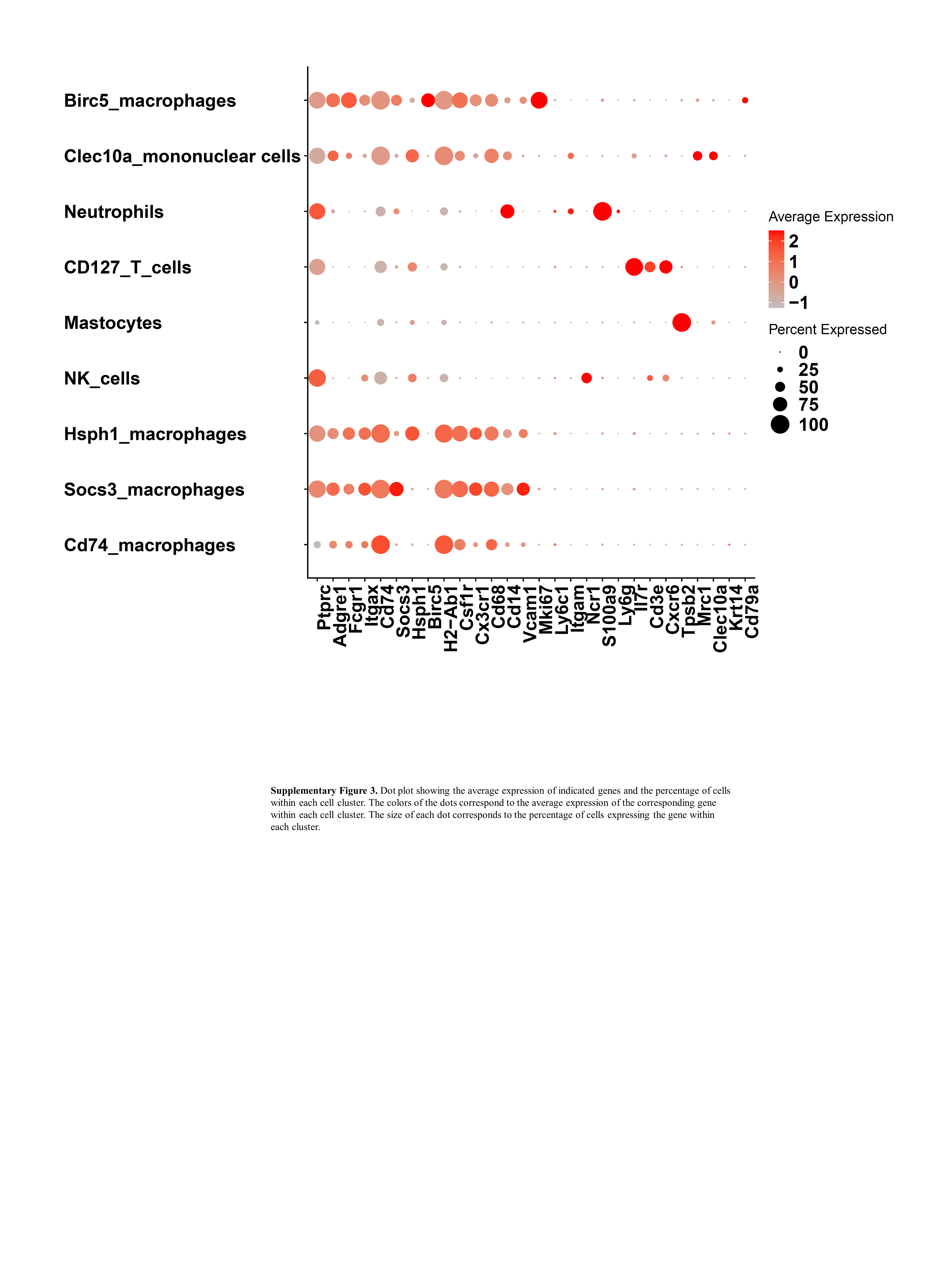

Supplement: Supplementary Figure 3 — Dot plot showing the average expression of indicated genes and the percentage of cells within each cell cluster. The colors of the dots correspond to the average expression of the corresponding gene within each cell cluster. The size of each dot corresponds to the percentage of cells expressing the gene within each cluster. [file Image_3.tif]

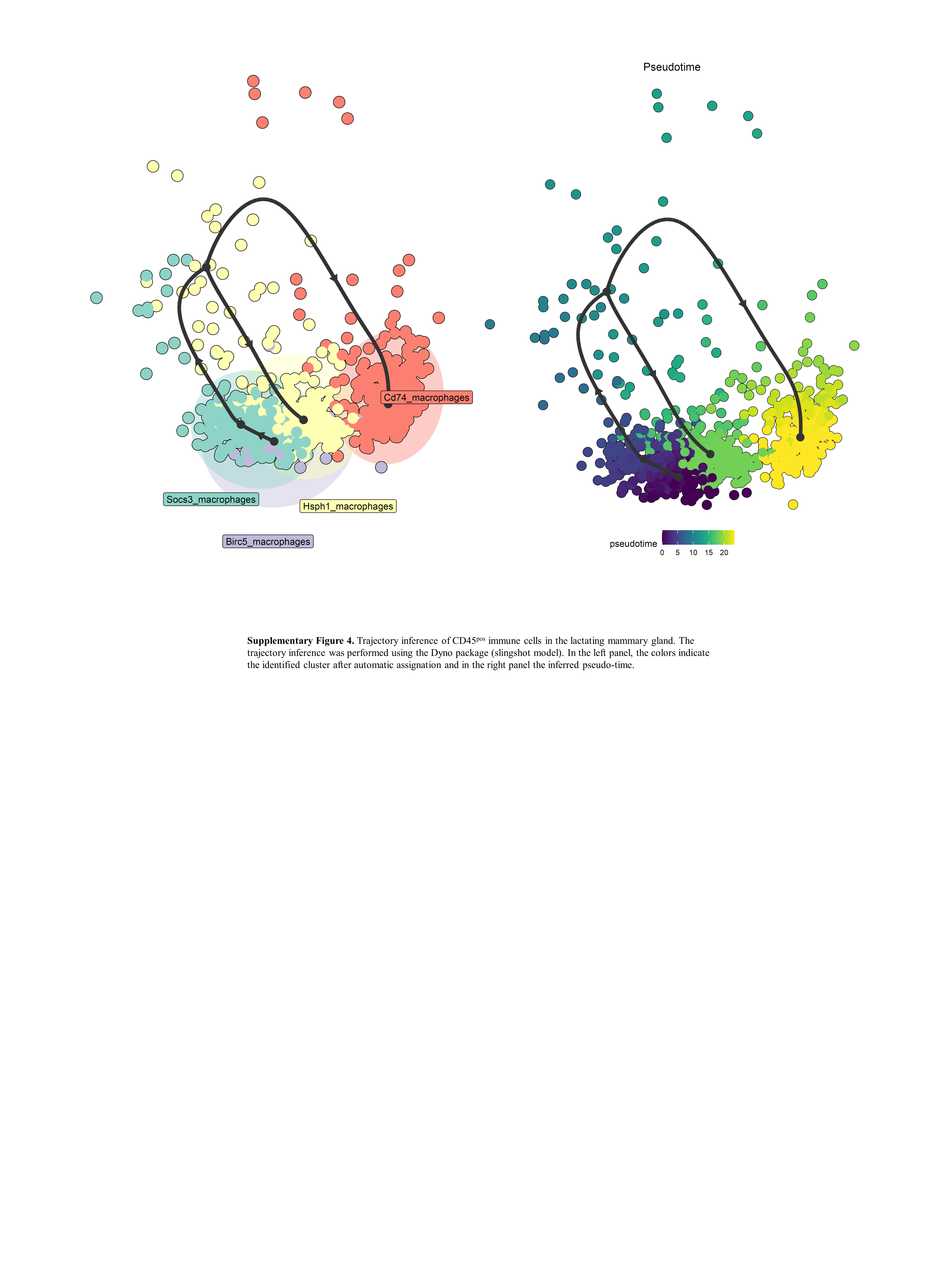

Supplement: Supplementary Figure 4 — Trajectory inference of CD45pos immune cells in the lactating mammary gland. The trajectory inference was performed using the Dyno package (slingshot model). In the left panel, the colors indicate the identified cluster after automatic assignation and in the right panel the inferred pseudo-time. [file Image_4.tif]
